# Risk of oral candidiasis: profile analysis of patients admitted to the dermatology clinic of a tertiary hospital in southeast of Brazil

**DOI:** 10.1590/S1678-9946202567054

**Published:** 2025-08-18

**Authors:** Ana Maria Hoyos Cadavid, Viviane Mazo Favero Gimenes, Vera Lúcia Teixeira de Freitas, Sonia Cristina Cavalcante, Lumena Pereira Machado Siqueira, Caroline Evelin Moraes Palomar, Ricardo Spina Nunes, Marcello Menta Simonsen Nico, Silvia Vanessa Lourenço

**Affiliations:** 1 Universidad de Antioquia, Facultad de Odontología, Departamento de Estomatología, Medellín, Antioquia, Colombia; 2 Universidade de São Paulo, Faculdade de Odontologia, Departamento de Estomatologia, São Paulo, São Paulo, Brazil; 3 Universidade de São Paulo, Faculdade de Medicina, Hospital das Clínicas, Laboratório de Micologia Médica (LIM-53), São Paulo, São Paulo, Brazil; 4 Universidade de São Paulo, Faculdade de Medicina, Instituto de Medicina Tropical de São Paulo, São Paulo, São Paulo, Brazil; 5 Universidade de São Paulo, Faculdade de Medicina, Departamento de Dermatologia, São Paulo, São Paulo, Brazil; 6 Universidade de São Paulo, Faculdade de Medicina, Departamento de Infectologia e Medicina Tropical, São Paulo, São Paulo, Brazil; 7 Universidade de São Paulo, Faculdade de Medicina, Hospital das Clínicas, Laboratório de Imunopatologia (LIM-06), São Paulo, São Paulo, Brazil

**Keywords:** Oral candidiasis, Immunocompromised patients, Candida albicans, *Candida* non albicans, ODDS ratio

## Abstract

The *Candida* genus colonizes the oral mucosa of immunocompetent individuals and healthy people, which is maintained by the innate immune system. However, any disturbance in this relationship, such as immunodepression, can turn this normally harmless yeast into a dangerous pathogen. This study evaluates the prevalence and risk factors for oral candidiasis (OC) among patients hospitalized in the dermatology department of a tertiary public hospital and identifies the *Candida* species involved. This cross-sectional study involves 240 patients. Oral candidiasis was diagnosed via clinical evaluation and mycological examination, with species confirmed using MALDI-TOF mass spectrometry. The prevalence of *Candida* species was 32.1%, in which *C. albicans* was the most common (92.1% of OC cases), followed by *Nakaseomyces glabrata, Pichia kudriavzevii, C. tropicalis*, and *C. parapsilosis.* Univariate analysis indicated that aging, use of oral prostheses, need for dental intervention, immunosuppression, and autoimmune diseases increase the risk of candidiasis. Multivariate analysis confirmed that aging, necessity for dental treatment, and immunosuppression were in 80% of OC cases. Given the great prevalence of oral candidiasis in hospitalized patients, dentists need to assess them for oral candidiasis and provide information on oral hygiene and healthy practices. Although *C. albicans* is the main microorganism responsible for these infections, other species have also been identified, highlighting the need for immediate identification and awareness of risk factors.

## INTRODUCTION

*C. albicans* is a ubiquitous commensal organism and is easily isolated from the oral cavities of healthy individuals. In fact, up to 80% of the general population is asymptomatic. *C. albicans* also asymptomatically colonizes the gastrointestinal and reproductive tracts of healthy individuals, where its proliferation is controlled by the immune system of the host^1,2^.

Local and systemic alterations in the host are associated with the risk of developing oral candidiasis (OC). Local changes include using orthodontic and prosthetic devices, poor oral hygiene, oral biofilm accumulation, hyposalivation, topical corticosteroid therapy, smoking, and altered physical integrity of the skin and mucosa. Systemic changes involve aging, broad-spectrum antibiotics, HIV and AIDS, and systemic diseases that compromise the immune system^1^.

Such infections can range from superficial skin and mucous membrane lesions to systemic infections that spread through the bloodstream, which affect different body organs. Candidiasis accounts for approximately 80% of all fungal infections^3^, and its increase in prevalence is related to diseases that suppress the immune system and the widespread use of immunosuppressive therapies^1-3^.

*C. albicans,* a pleomorphic fungus, can alternate between yeast-like and filamentous types, a critical element in the species’ pathogenesis. Other virulence factors involve the expression of adhesins and invasins on the cell surface, the formation of biofilms, and the secretion of hydrolytic enzymes^4^. These factors enable them to adapt to a variety of physiological extremes.

The diagnosis of OC occurs by finding a lesion compatible on clinical examination, diagnostic mycological by detection of blastoconidia, pseudohyphae, and true hyphae on direct mycological examination (DME) for better response to antifungal treatment, and exclusion of other possible diseases^5^.

Although *C. albicans* is still the most common cause of OC (50% to 80%), non-albicans *Candida* species (NACs) have gained importance in clinical context by increasing significantly in the last two decades^6-9^.

Identification of NACs has been relevant since most are less susceptible to azole drugs or show resistance to the available antifungal agents. These strains show different degrees of virulence^8,10-12^.

This study aims to evaluate the prevalence and risk factors for OC and to identify the *Candida species* isolated in a cohort of patients hospitalized in the dermatology department of a public tertiary hospital in Sao Paulo, Brazil.

## MATERIALS AND METHODS

### Patients

This study was conducted at the dermatology ward service of the Hospital das Clinicas, Medical School, University of Sao Paulo, Brazil. The sample included 240 patients admitted to the service from July 2017 to July 2019, without exclusion criteria regarding gender or age.

We collected detailed demographic information, data on medical conditions, immunosuppression status, diabetes mellitus diagnosis, the use of dental prostheses, the need for dental interventions, and smoking and drinking habits.

### Ethics

The study was rigorously reviewed and approved by the Ethics Committee under protocol Nº 2.018.627 (04/17/2017). The participants signed an informed consent form after receiving comprehensive information.

### Oral candidiasis

The diagnosis of oral candidiasis (OC) was based on the presence of compatible oral manifestations on clinical examination (n=67), which were associated with the detection of pseudohyphae and blastoconidia in the Direct Mycological Examination (DME). In this group also included patients taking medication for OC (10). A total of 77 cases were diagnosed with OC.

Isolation of *Candida* sp. in culture without oral signs and negative direct examination was considered colonization (Nonoral Candidiasis — NOC) except for patients undergoing OC treatment.

### Mycological diagnosis

All 240 patients underwent an oral mucosal examination. A sample from the oral mucosa was collected from the surface of the tongue, palate, or labial commissure using a sterile swab, irrespective of oral lesions due to a systemic disease or a clinical manifestation of oral candidiasis. Then, they were used for direct mycological exam.

Samples from 132 patients were cultured: 67 samples were collected from patients with clinical lesions consistent with OC, whereas 65 samples were randomly selected from 173 patients without signs of clinical lesions.

The selected samples were inoculated onto Sabouraud dextrose agar (SDA, DIFCO, USA) and incubated for 48 h at 30 °C. Positive samples were plated by exhaustion on CHROMagar Candida medium and incubated at 30 °C to obtain pure culture based on the color of the colonies. Phenotypic identification occurs by morphological, biochemical, and thermal tolerance^13^. The thermal tolerance test differentiated *C. albicans* and *C. dubliniensis* ^13^.

### MALDI-TOF

*Candida* sp. strains were identified using MALDI-TOF mass spectrometry. After each sample was planted in SAB for 48 h and incubated at 30 °C, a 10-µL loop was extracted. After adding 900 µL of 100% ethanol and 300 µL of sterile distilled water, the mixture was centrifuged for 2 min at 1300 rpm. The precipitate was left to dry at room temperature while the supernatant was disposed.

The dry pellet was then mixed in a vortex with 50 µL of 70% formic acid and 50 µL of acetonitrile, then centrifuged for 2 min. On a particular steel plate, one microliter of the supernatant was applied in quadruplicate at room temperature. One microliter of the HCCA matrix (α cyano-4-hydroxycinnamic acid) was then dissolved^14^.

The mass spectrometry analysis by MALDI-TOF was performed on a Bruker 3.1, comparing the resulting spectra with a database of reference spectra, expressed in log values from 0 to 3.

### Statistical analysis

All 240 cases were considered for the statistical analysis: 77 OC (67 patients with active lesion plus 10 patients under treatment) versus 163 NOC.

After an initial analysis of the patients’ data, exploratory techniques were used to identify distribution patterns, and trends in the main variables, univariate and multivariate logistic regression analysis was conducted.

The multivariate models had been tested for the multicollinearity of the independent variables using the Variance Inflation Factor (VIF). No multicollinearity was observed between the variables integrated into the binary logistic regression model.

To study the multivariate associations between independent variables (age, prosthesis, and immunosuppression) and the prevalence of OC, odds ratios (ORs) were estimated using a hierarchized logistic regression model. The results were expressed as ORs with their corresponding 95%CI for OC.

The statistical program used for this analysis was SPSS (version 24.0, IBM, New York, NY, USA).

## RESULTS

### Demographic profile

Overall, 240 patients admitted to the dermatology ward aged 1 to 89 years were included in this study. The mean age of the participants was 48.2±20.56. Most (63.3%) patients were over 40 years old and Caucasian (70.4%), and 57.1% of the participants were female.

About 30% of the patients had some type of immunosuppression. Of those, 92 required dental treatment, and 32.5% wore prostheses. Regarding personal habits, 37.4% reported being smokers, and 21.1% drinking alcoholic beverages.

### OC diagnosis and Candida sp. Identification

Of the 240 patients, 32.1% (77) were diagnosed with OC; 67 patients were diagnosed via clinical examination and DME, 59 of those were associated with pseudohyphae and blastoconidia on the DME test. Ten patients had previously been diagnosed in other services or clinics, were on antifungal therapy, and no longer had lesions or positive DME tests.

In all 67 cases, the clinical type of OC was classifiable, including four samples from patients being treated with nystatin, who still had clinical signs compatible with OC, three had the erythematous and one had pseudomembranous types (Table 1).

**Table 1:** Diagnosis of oral candidiasis.

Characteristic % (N)	Oral injury		p-value
	No 70.0 (163)	Yes 30.0 (77)	
**Age** ≥ 41 years	60.1 (163)	79.2 (77)	0.003*
**Sex** F	54.0 (163)	63.6 (77)	0.159*
**White** Y	71.2 (163)	68.8 (77)	0.829*
**Dental treatment** Y	21.5 (163)	75.3 (77)	<0.000*
**Dental prosthesis** Y	25.8 (163)	48.1 (77)	0.001*
**Smoker Y**	33.8 (142)	58.8 (68)	0.001*
**Alcoholic Y**	17.6 (142)	24.3 (70)	0.251*
**Oral injury description**		**N=67**	
Erythematous	NA	56.7	NA
Pseudomembranous		35.8	
Angular Cheilitis		7.5	
**DME**	3.7 (163)	76.6 (77)	<0.000*
**Antifungal**	0.0 (163)	13.0 (77)	NA
N = Total of cases; F = Female; Y = Yes; NA = Not applicable; DME = Direct mycological examination; *Pearson's chi-square.			

The erythematous type was the most prevalent in over 50% of OC cases, followed by pseudomembranous and angular cheilitis (Table 1). The palate was the most common site of infection, followed by the tongue and labial commissure

A total of 132 samples were cultured, of these, 67 were from patients with clinical signs of oral candidiasis (OC), while the remaining 65 samples were randomly selected from patients without clinical signs. Table 1 shows the results.

**Figure 1:**
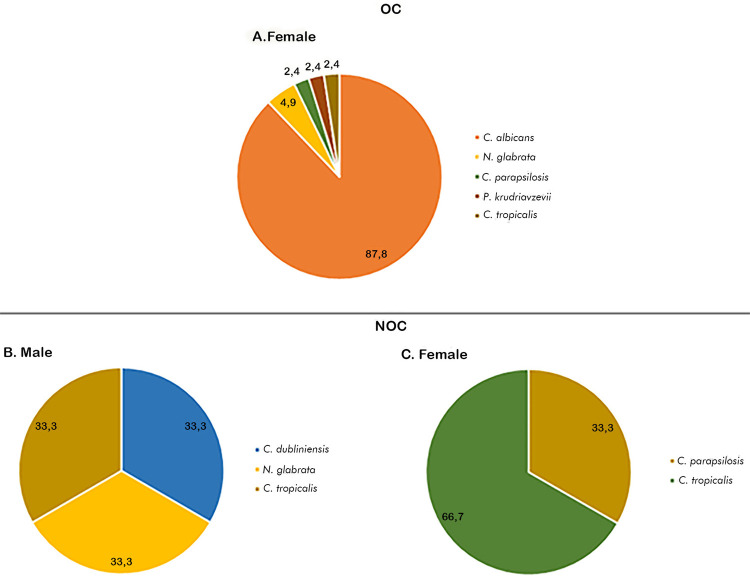
Frequency of species isolated by culture according to sex and diagnosis.

After isolation by culture, morphological and biochemical analyses, and MALDI TOF confirmation, five *Candida* species were identified in patients with OC diagnosis. *C. albicans* was the most common (58 out of 63 samples), followed by *Nakaseomyces glabrata* (*C. glabrata), Pichia krudriavzevii* (*C. kruzei)*, and *C. tropicalis*, *C. parapsilosis* was identified in a recently treated patient with no compatible clinical picture. Five yeast samples could not be identified due to contamination (Table 1). NCAs were observed in 12.2% of females with OC, with a mean age of 46 years (Figure 1A), whereas CAs were identified in patients of both sexes with a mean age of 54 years.

The following were isolated in six samples from the 64 patients who had no clinical signs compatible with candidiasis or positive DME test: *C. parapsilosis* (2), *C. tropicalis* (2), *N. glabrata* (1), and *C. dubliniensis* (1) (Table 1). These results were considered colonization (NOC) (Table 1, Figures 1B and 1C).

These findings showed a high prevalence of *Candida albicans* in patients diagnosed with OC. The results also highlight NCAs in female patients with oral lesions.

**Figure 2:**
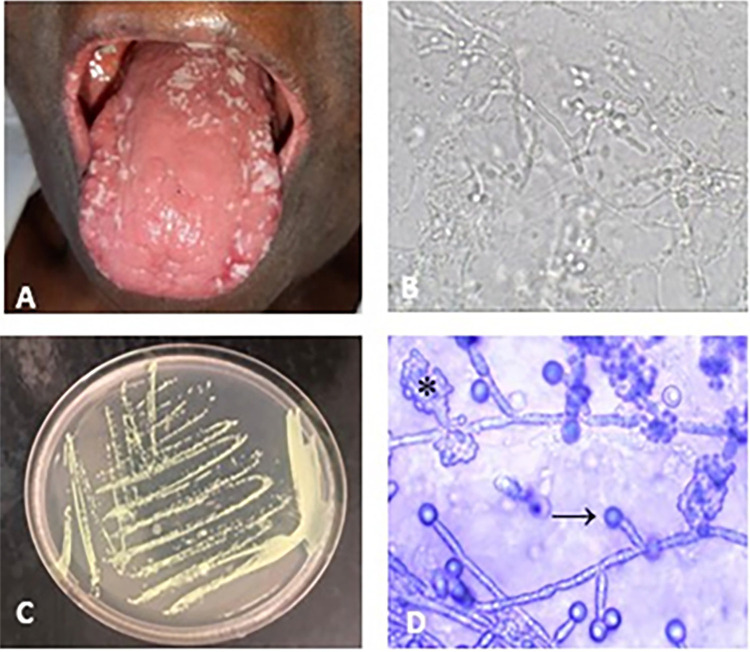
Clinical features and microbiological findings of fungal characterization methods: (A) Clinical presentation of pseudomembranous candidiasis, showing as widespread nonadherent white plaques on the dorsal tongue; (B) Direct examination, revealing yeast hyphae; (C) Identification of fungal species by CHROMagar, with *Candida albicans* in green; (D) Microculture of chlamydoconidia (arrow) and blastoconidia (asterisk) within fungal clusters.

Figure 2 shows an example of the clinical appearance of OC in the pseudomembranous type and the microscopic characteristics of the yeast on direct mycological examination and in microculture; it is also possible to observe the growth of *C. albicans* in CHROMagar medium.

### Demographic profile

Table 2 summarizes the distribution of colonization and OC cases according to demographic variables and personal habits.

**Table 2:** Demografic profile of patients admitted to the dermatology ward.

Characteristic % (N)	OC		p-value
	No 67.9 (163)	Yes 32.1 (77)	
**Age** ≥ 41 years	60.1 (163)	79.2 (77)	0.003*
**Sex** F	54.0 (163)	63.6 (77)	0.159*
**White** Y	71.2 (163)	68.8 (77)	0.829*
**Dental treatment** Y	21.5 (163)	75.3 (77)	<0.000*
**Dental prosthesis** Y	25.8 (163)	48.1 (77)	0.001*
**Smoker Y**	33.8 (142)	58.8 (68)	0.001*
**Alcoholic Y**	17.6 (142)	24.3 (70)	0.251*

N = Total of cases; F = Female; Y = Yes; NA = Not applicable; DME = Direct mycological examination; *Pearson’s chi-square.

Most OC patients were over 40 years of age, required dental treatment, and smoked, and 48% of OC patients wore dental prostheses. All these variables showed statistically significant differences among NOC patients.

Although women accounted for most of the patients with OC, the difference was not significant.

### Clinical profile

Serious systemic diseases were the reason for hospitalization in all patients. Inflammatory diseases were the most frequent cause of hospitalization (38% of the patients), followed by autoimmune diseases (about 30% of the patients). Other conditions, such as infections, neoplasms, and hereditary diseases, were part of the clinical profile of the patients.

Supplementary Table S1 shows that oral candidiasis was mainly associated with oral lesions caused by autoimmune and hereditary diseases (47% of cases of OC). These patients had the following underlying diseases: pemphigus, psoriasis, systemic lupus erythematosus, bullous pemphigoid, lichen simplex chronicus, Sjogren’s syndrome, chronic mucocutaneous candidiasis, Rendu Osler Weber syndrome, and epidermolysis bullosa.

### Analysis of risk factors for OC

Overall, 240 cases— 163 without OC and 77 with OC—were used to analyze risk factors for OC. The prevalence of OC was 32.1%.

### Univariate analysis

Table 3 shows the results of the univariate analysis of risk factors, including demographic data, clinical data, and personal habits (smoking and alcohol intake).

**Table 3:** Univariate analysis of potential risk factors for developing OC.

Independent characteristic	N	OR	95%CI		p-value
			Lower -	Upper	
Age ≥ 41 years	240	2.529	1.342 -	4.765	0.004
**White**	240	0.895	0.496 -	1.613	0.712
Female	240	1.491	0.854 -	2.604	0.160
Dental treatment	240	11.164	5.893 -	21.149	<0.001
Dental prosthesis	240	2.665	1.509 -	4.705	<0.001
Autoimmune or Hereditary diseases	240	2.302	1.310 -	4.048	0.004
**Diabetes Mellitus**	233	1.188	0.650 -	2.173	0.575
Immunodepressed	240	4.259	2.365 -	7.668	<0.001
Smoker	214	1.567	0.877 -	2.799	0.130
**Alcoholic**	**213**	**1.630**	**0.829 -**	**3.205**	**0.156**

Preliminary univariate analyses showed potential associations between the development of OC and patients aged > 40 who required dental care, wore dentures, were immunosuppressed, and had autoimmune or hereditary diseases.

The risk of developing OC was higher in patients older than 40 years (OR 2.529; p=0.004), and 11 times higher in patients who required dental treatment, which suggests a lack of oral hygiene care. Wearing a prosthesis is also a risk factor; 42.9% (33/77) of patients who wore prostheses were diagnosed with OC, increasing the chance of *Candida* infection by 2.6 times.

Among the associated clinical factors, immunosuppressed patients have a high risk (OR 4.259; p<0.001). Patients with autoimmune and hereditary diseases, especially those associated with damage to the oral mucosa, were also at higher risk (OR 2.3; p=0.004)

There were no significant differences regarding sex, ethnicity (Caucasian), and presence of diabetes. The same was true for smokers and alcohol users.

### Multivariate analysis

Multivariate logistic regression analysis examined the association between independent variables that had the lowest p-values (less than 0.2). Table 3 shows these variables in bold.

Various associations between independent variables were tested. The model including dental treatment, age ≥41 years, sex (female), and immunosuppression in multivariate logistic regression analysis predicted 80.0% of OC cases and was considered the best association (Table 4).

**Table 4:** Multivariate analysis of potential risk factors for developing OC.

Independent Characteristic	OR	95%CI		p-value
		Lower -	Upper	
**Age ≥ 41 years**	3.546	1.598 -	7.866	0.002
**Female**	1.591	0.791 -	3.199	0.193
**Imunossupressed**	4.251	2.062 -	8.765	<0.001
**Dental treatment**	10.955	5.501 -	21.977	<0.001

As shown in Table 4, significant associations were observed for age (OR 3.546; CI 1.598-7.866), immunosuppression (OR 4.251; CI 2.062-8.765), and the need for dental treatment (OR 10.995; CI 5.501-21.977).

Univariate analysis did not show an increased risk of OC in women.

## DISCUSSION

*Candida spp.* is one of the major opportunistic pathogenic fungi commonly found in the physiological microbiota^1,2^. The ability to colonize various anatomical sites and transform from commensal to pathogen depends on its virulence and the environmental and local conditions that modify the microenvironment in the oral cavity^1,2^, especially in patients with specific conditions, such as smoking, decreased vertical dimension, and hyposalivation^15^, which are common in our patient group.

In this study, the prevalence of OC was 32.1%. This data was similar to the results presented in other reports: one study, with a population similar to ours, found a prevalence of with 30%^8^; and one study involving children, from newborns to 12 years of age, found a prevalence of 34%^16^.

Another report showed a prevalence of 16.3% (n=141) in medium and high-complexity hospital patients, and the diagnostic criteria were based on clinical assessment. Of the 141 patients, 63 had no systemic diseases, 38 had diabetes, and 71 had neoplasms^17^. This population differed from our study, in which inflammatory and autoimmune diseases were predominant.

A 2003 report showed a prevalence of 42.7% of OC, which is probably due to the long-term selection of older adults (average age 85) in geriatric clinics^18^.

This study shows that individuals over 40 year of age are 2.5 times more likely to develop oral *Candida sp.*, so age is a significant risk factor.

In a study involving 160,357 individuals, oral candidiasis (OC) was diagnosed in 9,769 patients. The study showed that the rate of *Candida* spp. infection increases with age^7^.

The flow of saliva decreases with aging. Saliva contains antifungal proteins, such as histatin-5, which prevent *C. albicans* from adhering to the oral mucosa of the host and play an essential role in controlling the fungal population^1,15,19^. Furthermore, innate and adaptive immune systems lose their effectiveness against pathogens as people age^20^.

Our study reported a 2.7 times higher risk of OC in patients who wore dentures, but several studies have demonstrated this significant relationship, with a prevalence of OC among denture wearers ranging from 56% to 83%^16,21-23^. One of the reasons is the formation of a relatively acidic and anaerobic microenvironment due to the use of oral prostheses, and the porosity of their acrylic surfaces provide viable conditions for the colonization of bacteria and fungi^21-23^. Inadequate hygiene also contributes to a microenvironment favorable to oral infection^18,22,23^.

The relatively acidic and anaerobic microenvironment created by oral prostheses and the porosity of their acrylic surfaces provide viable conditions for the colonization of bacteria and fungi^19,21^. In addition, inadequate hygiene contributes to a microenvironment favorable to oral infection^16,23,24^.

Nearly 40% of the participants and 75% of those with OC required dental care.. This variable proved to be an important predictive factor for the development of infection . According to a survey on the oral health of Brazilians conducted in 2010, 68.8% of relatively young adults (aged 35 to 44 years) required some dental prosthesis, most of which were partial dentures in one jaw, and 1.3% of cases required a total prosthesis in at least one jaw. Among older individuals aged 65-74 years, 23.9% of cases needed a total prosthesis in at least one jaw, and 15.4% needed a dual total prosthesis^25^. This study reveals the precariousness of oral health care and the inaccessibility of care and guidance from dental professionals in Brazil.

All 240 patients admitted to the dermatology unit had severe underlying diseases, in which 30% were immunosuppressed, another critical factor in the development of OC. These include autoimmune diseases (such as pemphigus, psoriasis, systemic lupus erythematous, bullous pemphigoid, lichen simplex chronicus, Sjogren’s syndrome), hereditary diseases (Chronic mucocutaneous candidiasis, Rendu Osler Weber syndrome, and epidermolysis bullosa), and inflammatory diseases (erythroderma, dermatomyositis, adverse cutaneous drug reactions, Crohn’s disease).

Oral lesions can be the first and occasionally the only manifestation of immune-mediated diseases affecting the skin and mucous membranes. Autoantibodies directed against structural compounds of skin and oral mucosa, and inflammatory infiltrates damage the oral mucosa to varying degrees^26-30^.Infections can progress rapidly in patients with autoimmune or inflammatory diseases due to immunosuppression associated with the disease or therapy^30^.

Regarding pathogen identification, *C. albicans* predominated in 92.1% of OC cases. Other less common species, including *N. glabrata*, *C. tropicalis*, *P. krudriavzevii*, *C. parapsilosis*, and *C. dubliniensis*, were also identified in our study. Notably, NCA species were detected as causative agents of OC only in females, and they were also identified as a commensal microorganism in 9.4% of NOC patients in both sexes. Cheng *et al*.^31^ showed that patients under 65 years of age are at a higher risk of developing Candidemia caused by NCAs. In this report, the five women with OC had a lower mean age than those who had isolated ACs .

*C. albicans* continues to be responsible for more than 50% of human yeast infections^8,11,15,29^. However, in the last decade, the number of infections due to NCAC species has increased significantly^6-9,32^. Since different *Candida* species show variable resistance patterns, rapid identification to species level is a substantial prerequisite for convenient management of antifungal therapy, especially if antifungal susceptibility testing is not accessible^1^.

Most oral infections caused by this fungus are typically superficial but can progress to systemic infection if left untreated, particularly in immunosuppressed patients.^29^

*N. glabrata* has increased significantly as an infectious agent, particularly in hospitals^33,34^. It can become resistant to fluconazole and echinocandins and is more common in older adults^7,35,36^, all patients were over 60 years in this study.

*C. tropicalis* is resistant to 5-fluorocytosine and causes candidemia in adults, especially in patients with malignancies and diabetes mellitus (DM)^37^. In our study, two out of three patients had DM, and none was diagnosed with a malignant neoplasm.

The multi-resistance phenotype of *P. kudriavzevii* (mainly fluconazole, amphotericin B, and 5-fluorocytosine) complicates its treatment, especially in immunocompromised patients^36^.

*C. parapsilosis* is the most common species in bloodstream infections. In our sample, the three patients were under 20 years of age; none had a history of transplantation, and one used a central catheter. The clinical isolates of this species are susceptible to amphotericin B and azole derivatives^37^.

Since its characterization in 1995, *C. dubliniensis* has attracted the attention of researchers because this yeast has phenotypic and genotypic characteristics similar to *C. albicans.* Phenotypic tests are useful for the presumptive identification of *C. dubliniensis,* but they do not provide a definitive identification; on the contrary, molecular methods provide a conclusive identification, but they are difficult to perform, expensive, and require specific equipment^37^.

*C. dubliniensis* is commonly associated with HIV-positive patients and patients with DM and can quickly develop resistance to azoles^6,37^. The only patient who tested positive for this species did not show any of the above pathologies.

Recently, there has been a marked increase in morbidity and mortality associated with *Candida* infections and decreased susceptibility to commonly used antifungal agents^6,33-35^; thus, it is necessary to understand local trends and species distribution among high-risk patients.

The change in the pattern of Candida infections in high-risk patients with a predominance of NCA species has often been attributed to previous exposure to antifungals, broad-spectrum antibiotics, chemotherapy, invasive devices, surgeries, prolonged hospitalizations, ICU admissions, and neutropenia. Additionally, immunosuppressive conditions and comorbidities are frequently found in high-risk patients and play a significant role in this change^38^.

Rapid identification of these species is necessary due to the higher mortality rates associated with these infections, particularly in high-risk patients^2,3^.

Timely and accurate identification of these species in the hospital setting is essential to provide specific therapy. The new MALDI-TOF system provided rapid and accurate species identification in our study, which is essential for individualization of therapy, reduction of resistant strains, and implementation of effective infection control measures^37,38^. However, its high cost makes it difficult to use in routine practice.

Chromagar is an inexpensive and simple method for identifying common *Candida* species, particularly in areas with limited resources. This also enables rapid diagnosis and epidemiological surveillance in high-risk units, however, it remains insensitive and unable to identify all species^39^.

## CONCLUSION

Oral candidiasis was significantly associated with age, use of dentures, need for dental care, and autoimmune and hereditary diseases. Assessment of hospitalized patients by dentists is encouraged to identify signs of oral candidiasis and to educate patients on better oral hygiene and healthy lifestyles.

Non-albicans *Candida* species are becoming increasingly common in hospital settings, particularly in patients with critical illnesses like our sample. Such species require rapid and accurate identification, and an understanding of the local trends, distribution, and risk factors associated with them so that the best therapeutic option can be chosen.

## Appendix 1

Supplementary Material available from: https://data.scielo.org/dataset.xhtml?persistentId=doi:10.48331/SCIELODATA.BDY7ZH
